# Factors Affecting Patient Satisfaction in Dermatology Clinics in Saudi Arabia

**DOI:** 10.7759/cureus.32193

**Published:** 2022-12-05

**Authors:** Fawwaz F Al Shammrie, Wael S Alanazi, Mohammad A Alduheim, Abdullah A Alenazi, Yasmin S Alhamazani, Fatimah Y Alkhuraisi, Afnan H Alshammari, Rakan A Alshammari, Basil A Alshammari, Lenah H AlMarzouk

**Affiliations:** 1 Dermatology, University of Hail College of Medicine, Hail, SAU; 2 Dermatology, King Salman Specialist Hospital, Hail, SAU; 3 Plastic and Reconstructive Surgery, King Salman Specialist Hospital, Hail, SAU; 4 Medicine, University of Hail College of Medicine, Hail, SAU; 5 Dermatology, King Fahad General Hospital, Madinah, SAU; 6 General Practice, King Salman Specialist Hospital, Hail, SAU; 7 Medicine, Alfaisal University College of Medicine, Riyadh, SAU

**Keywords:** dermatology clinics, outpatient care, satisfaction factors, dermatology clinics satisfaction, patient satisfaction

## Abstract

Background

A methodical approach to health services called “quality of care” places an emphasis on both interpersonal and technical competence in the delivery of medical care. One of the key elements of high-quality care is the client's or patient's satisfaction, which includes treating them with respect, being aware of their needs, and meeting those needs. Dermatologists are forced to expand service availability and respond to patients' needs and desires as required through satisfaction surveys as a result of an increase in the number of insured patients and demand for services.

Methods

A cross-sectional study was conducted in Saudi Arabia to measure patient satisfaction with the medical care provided to patients in dermatology clinics affiliated with the government and private hospitals by recording the responses received from a questionnaire assessing the doctor’s attitude, such as whether the dermatologist was punctual and had the patient’s medical records. The modified Arabic language digitally administered questionnaire with 17 closed-ended items was used to collect data from respondents.

Results

In total, 1002 patients, including 70.76% females and 29.24% males, participated in the study; 83.33% of patients visited a dermatology clinic of which 74.61% visited private clinics, and the disease had already developed in 64.55% of patients. A 5-point Likert scale with six statements was used to measure patient satisfaction with the visiting doctor. Overall, the mean score was very high (4.41 ± 0.92).

Conclusion

According to the results of this study, respect for patients’ wishes, emotional support, and physical comfort appear to be the most crucial factors in determining patient happiness. Healthcare systems should strive to achieve a balance in services that not only offer clinically effective care but are also perceived by the patients as acceptable and beneficial. This is true regardless of demographic status.

## Introduction

Skin diseases require regular follow-up appointments and long-term care. Chronic skin diseases often reduce the quality of life of patients. Thus, patients with psoriasis have the same symptoms as those with cancer, depression, heart disease, and diabetes in terms of quality of life [1]. Despite the importance of the dermatologist-patient relationship, patient satisfaction is rarely studied in the context of chronic skin diseases [2]. An important indicator of the quality of care is patient satisfaction, and this can be assessed by defining patient satisfaction with healthcare providers [3]. Patient satisfaction is important because it may lead to better adherence to therapy and, therefore, better clinical outcomes [4]. Dermatology frequently assesses the quality of life of patients with chronic skin illnesses, but patient satisfaction is rarely examined despite the doctor-patient interaction's significance in this setting. We strive hard to satisfy our patients’ expectations when providing care for them. Although doctors try their best, a patient’s impression of the quality of doctor consultations could differ [5]. There are not many studies assessing patient satisfaction in dermatology clinics. Patient satisfaction surveys will become more prominent, and hospitals and other providers will use patient responses to highlight the high caliber of their services. Patient satisfaction is very important in determining whether or not patients follow their treatment plan and visit their doctor [6]. It is critical to recognize the primary variables affecting patient satisfaction in order to enhance the way patients perceive the quality of their care. Infrastructures, access to care, interpersonal skills of staff, and patient-related factors such as sociodemographics of patients, disease severity, and health-related quality of life are a few examples of provider-related factors. Given the effects of skin illnesses on patients’ lives, assessment of the quality of life can be particularly significant in dermatology [7]. Furthermore, studies have demonstrated that health-related quality of life measurements are not always associated with a doctor’s assessment of the clinical severity of a skin illness [8].

## Materials and methods

Objectives

This study aims to measure patient satisfaction with medical care provided to patients in dermatology clinics affiliated with the government and private hospitals in Saudi Arabia by answering a questionnaire assessing their physician’s attitude such as whether the dermatologist was punctual, knew the patient’s medical history, discussed health queries and concerns, listened carefully to the patient, showed respect for what the patient had to say, explained information clearly, spent enough time with the patient, and aimed for quality improvement and optimization of the physician-patient relationship.

Study design and sample

This cross-sectional study was performed on male and female patients attending dermatology clinics affiliated with government and private hospitals in Saudi Arabia. The Research Ethics Committee at the University of Hail approved the study with an approval number H-2022-322.

Data collection

The modified electronic administered questionnaire in Arabic was used for data collection from respondents that contained 17 closed-ended questions. It was distributed through social media channels from July to October 2022 targeting all patients attending dermatology clinics affiliated with government and private hospitals in Saudi Arabia. The questionnaire was first given to 10 randomly selected participants. The purpose of this pilot testing was to assess the clarity of questions and identify ambiguous or lengthy questions, in addition to testing its applicability.

Data analysis

The data were analyzed using SPSS Statistics, version 25.0 (IBM Corp., Armonk, NY). The frequencies, percentage, mean, and standard deviation were determined to describe the items and domains. The independent t-test and one-way ANOVA were conducted to test the differences in the visiting, examination, and services in terms of sociodemographic variables with post hoc consideration to test multiple comparisons. A p-value less than 0.05 was considered statistically significant.

## Results

As shown in Table 1, a total of 1002 patients participated in the study including 70.76% females and 29.24% males; the age was classified into five categories with an advantage for patients aged 20-30 years comprising 33.53%, followed by 31-40 years comprising 29.34%, and then 41-50 years comprising 22.75%. More than three-quarters held a bachelor’s degree; the study also covered five regions with an advantage for the central region, followed by northern and eastern with approximately 26% each.

**Table 1:** Demographic information (N = 1002)

Variable		N (%)
Gender	Male	293 (29.24)
	Female	709 (70.76)
Age		
	Less than 20	84 (8.38)
	20–30	336 (33.53)
	31–40	294 (29.34)
	41–50	228 (22.75)
	More than 50	60 (5.99)
Education		
	High school or less	188 (18.76)
	Bachelor	756 (75.45)
	Postgraduate	58 (5.79)
Region		
	Central	339 (33.83)
	Western	40 (3.99)
	Eastern	263 (26.25)
	Southern	95 (9.48)
	Northern	265 (26.45)

As shown in Table 2, 83.33% had visited a dermatology clinic, with 74.61% visiting a private clinic, of which 64.55% visited with a disease and 35.45% visited for cosmetic reasons. A total of 59.52% of female doctors and 40.48% of male doctors served in the clinic.

**Table 2:** Visiting information (N = 835)

Variable		N (%)
Have you ever visited a dermatology clinic?		
	Yes	835 (83.33)
	No	167 (16.67)
Type of clinic		
	Government	212 (25.39)
	Private	623 (74.61)
Reason for visiting the clinic		
	Cosmetic reason	296 (35.45)
	Disease	539 (64.55)
Doctor’s gender		
	Male	338 (40.48)
	Female	497 (59.52)

As shown in Table 3, the distribution of patient satisfaction with the visiting doctor was measured using a 5-point Likert scale with six statements. The overall mean score showed a very high level of satisfaction (4.41 ± 0.92); the statement ranged between 4.42 ± 0.97 and 4.11 ± 0.95.

**Table 3:** The distribution of patient satisfaction with the visiting doctor (N = 835) Total mean score = 4.41 ± 0.92, a very high level of satisfaction

Statement	Dissatisfied	Satisfied	Neutral	Satisfied	Very satisfied	Mean ± SD
Was the time of appointment chosen for you?	18 (2.16)	26 (3.11)	49 (5.87)	342 (40.96)	400 (47.90)	4.29 ± 0.88
Was there a waiting time for the consultation?	23 (2.75)	41 (4.91)	73 (8.74)	384 (45.99)	314 (37.60)	4.11 ± 0.95
Was there an explanation from the dermatologist for the pathological condition?	21 (2.51)	40 (4.79)	41 (4.91)	197 (23.59)	536 (64.19)	4.42 ± 0.97
How satisfied are you with the duration of the discussion of the diseased condition?	22 (2.63)	54 (6.47)	60 (7.19)	286 (34.25)	413 (49.46)	4.21 ± 1.01
How satisfied are you with the discussion of the disease in terms of the information provided and methods of treatment?	25 (2.63)	50 (5.99)	47 (5.63)	195 (23.35)	518 (62.04)	4.35 ± 1.03
How satisfied are you with the dermatologist’s response to your questions?	13 (1.56)	40 (4.79)	52 (2.99)	216 (25.87)	514 (61.56)	4.41 ± 0.92

As shown in Table 4, the distribution of examination was presented; for 85.03% of patients the skin was examined with a very high level of satisfaction (4.56 ± 0.69); however, only for 13.38% of patients, pictures were taken for the purpose of skin examination and only for 6.76% of patients, a skin sample (biopsy) was taken. The satisfaction toward the service in terms of listening to your problem, respecting your decisions, and valuing your privacy was 4.48 ± 0.88.

**Table 4:** Examination information (N = 710)

Variable		N (%)
Has the skin been examined?		
	Yes	710 (85.03)
	No	125 (14.97)
How satisfied are you with the examination?		
	Very dissatisfied	3 (0.42)
	Dissatisfied	10 (1.41)
	Neutral	31 (4.37)
	Satisfied	207 (29.15)
	Very satisfied	459 (64.65)
Were pictures taken for the purpose of examination?		
	Yes	95 (13.38)
	No	615 (86.62)
Was a skin sample (biopsy) taken?		
	Yes	48 (6.76)
	No	662 (93.24)
What is your opinion about the doctor providing the service in terms of listening to your problem, respecting your decisions, and valuing your privacy?		
	Very dissatisfied	18 (2.16)
	Dissatisfied	20 (2.40)
	Neutral	53 (6.35)
	Satisfied	194 (23.26)
	Very satisfied	549 (65.83)

Table 5 shows independent t-test and one-way ANOVA were conducted to test the differences in visiting, examination, and services in terms of sociodemographic variables with post hoc consideration to test the multiple comparisons. Females were reported to have a higher level of satisfaction than males (p < 0.001).

**Table 5:** The distribution of satisfaction of visiting, examination, and services in terms of sociodemographic information (N = 1002) *p ≤ 0.05; **p ≤ 0.01; ***p ≤ 0.001

Variable		Satisfaction mean ± SD		
		Visiting	Examination	Service
Gender				
	Male	4.10 ± 1.01	4.36 ± 0.89	4.14 ± 1.09
	Female	4.37 ± 0.65	4.62 ± 0.60	4.59 ± 0.78
	p-value	p < 0.001***	0.002**	p < 0.001***
Age				
	Less than 20	4.46 ± 0.50	4.66 ± 0.56	4.78 ± 0.45
	20–30	4.38 ± 0.66	4.61 ± 0.59	4.60 ± 0.74
	31–40	4.26 ± 0.80	4.53 ± 0.80	4.39 ± 0.99
	41–50	4.24 ± 0.82	4.50 ± 0.72	4.34 ± 1.01
	More than 50	4.06 ± 0.94	4.42 ± 0.71	4.23 ± 0.97
	p-value	0.02*	0.25	p < 0.001***
	Post hoc	1 > 3,4,5 2 > 5	––	1 > 3,4,5 2 > 5
Education				
	High school or less	4.30 ± 0.85	4.56 ± 0.74	4.53 ± 0.92
	Bachelor	4.33 ± 0.71	4.58 ± 0.64	4.50 ± 0.83
	Postgraduate	4.19 ± 0.87	4.30 ± 0.99	4.11 ± 1.20
	p-value	0.42	0.04*	0.004**
	Post hoc	––	1 > 3 2 > 3	1 > 3 2 > 3
Region				
	Central	4.49 ± 0.43	4.68 ± 0.56	4.73 ± 0.65
	Western	4.19 ± 0.94	4.38 ± 0.86	4.32 ± 1.04
	Eastern	4.08 ± 0.93	4.50 ± 0.81	4.24 ± 1.09
	Southern	4.61 ± 0.34	4.76 ± 0.43	4.80 ± 0.43
	Northern	4.14 ± 0.91	4.33 ± 0.76	4.21 ± 0.94
	p-value	p < 0.001***	p < 0.001***	p < 0.001***
	Post hoc	1 > 3,4 4 > 3,5	1 > 5 4 > 5	1 > 3,5 4 > 3,5
Type of clinic				
	Government	4.10 ± 0.93	4.42 ± 0.87	4.26 ± 1.06
	Private	4.38 ± 0.66	4.61 ± 0.60	4.56 ± 0.80
	p-value	p < 0.001***	p < 0.001***	p < 0.001***
Reason for visiting the clinic				
	Cosmetic reason	4.33 ± 0.69	4.65 ± 0.55	4.53 ± 0.83
	Disease	4.30 ± 0.77	4.52 ± 0.74	4.45 ± 0.91
	p-value	0.579	0.020*	0.210
Gender (doctor)				
	Male	4.22 ± 0.89	4.51 ± 0.77	4.35 ± 0.99
	Female	4.37 ± 0.63	4.60 ± 0.62	4.57 ± 0.79
	p-value	0.008**	0.100	p < 0.001***

There were differences in satisfaction of visiting (p < 0.05) and service (p < 0.001) in terms of age with a disadvantage for patients less than 30 years, the post hoc test found whom significantly were more than all groups except 20-30 years (p < 0.01). The results indicated that young patients had higher satisfaction than older patients.

There were differences in satisfaction of examination (p < 0.05) and service (p < 0.001) in terms of education with a disadvantage for patients who completed postgraduation who were significantly lower than patients with high school or less education and bachelor education (p < 0.01).

There was a difference in satisfaction (visiting, examination, and service) in terms of the region with an advantage for patients from the southern region (p < 0.001). The southern region was higher than the eastern region for visiting and services and higher than the northern region for visiting, examination, and service (p < 0.001). In addition, the central region was higher than the northern region for examination and service and higher than the eastern for visiting and services (p < 0.001). Patients were more satisfied with private clinics than government clinics (p < 0.001); cosmetic reason was the purpose of examination (p < 0.05), and females exceeded males in visiting (p < 0.01) and service (p < 0.001).

## Discussion

In recent years, patients are increasingly viewed as consumers or customers by the healthcare system [9]. Measuring patient satisfaction with care on a regular basis has become very crucial for healthcare practitioners. Assessing the patient’s perspectives and determining whether they believe their requirements are satisfied are the key components of measuring patient satisfaction [9,10]. Patients can assess if they have received enough information and can assess the demeanor and attitudes of their doctors, but they are typically unable to accurately assess the veracity of a diagnosis or treatment plan. Fortunately, medical staff has direct control over the latter, making it feasible to improve patient satisfaction with the required effort [11].

In this study, dermatology clinics in Saudi Arabia were evaluated for overall patient satisfaction. In the skin examination, there was a very high degree of satisfaction (85.03%) with the overall quality of dermatology treatments. The range of values reported for this location is comparable to the level of patient satisfaction. It was discovered that females expressed greater levels of satisfaction with visits, examinations, and services than males. The prospective design of our study, the consistency of responses with those from other studies, and, in particular, the identification of key skills for doctors to learn, including paying attention to the patient’s quality of life, are the strong points of the study.

A thorough skin inspection is frequently required during a dermatologist visit, which is a crucial component of patient satisfaction, especially for people under 30 years and over 50 years of age. However, patients are occasionally reluctant to have a thorough skin examination due to time constraints, noticeable skin conditions such as psoriasis or atopic dermatitis, and changed perceptions of themselves. Other unique aspects of dermatological consultations, such as the use of a dermatoscopy or the performance of a skin biopsy, are variables for patient satisfaction in patients aged 50 and older. Therefore, in addition to age stratification, these technical skills were taken into account. Even in consultations at the hospital, a doctor’s interpersonal ability is the most crucial factor in determining patient happiness. Doctors’ judgments of the quality of life of patients in particular seem to be one of the most important determinants of patient satisfaction.

This study showed that more effort needs to be taken in government and private clinics on the interaction of staff or clerks with patients because both clinics had poor scores in this factor. In addition, communication needs to be improved. Clinicians must use extra caution to refrain from communicating with their patients in a frustrated or dismissive manner. Moreover, clinic delays should be handled efficiently, and their underlying causes should be identified. If it is determined that patients are to blame for delays, it might be helpful to identify those patients who consistently arrive late or do not show up at the clinic. Then, appointments for these patients may be fixed later in the day to reduce disruptions to the clinic schedule and daily patient flow.

In order to effectively treat dermatological conditions such as psoriasis and acne, several studies have emphasized the significance of patient-physician communication and have developed specific protocols. According to these protocols, the treatment should be suitable to the patient’s lifestyle, and patients should be provided with clear information, such as the anticipated time frame for improvement and the anticipated side effects [12]. Researchers studying about psoriasis patients have stressed the importance of patient involvement in treatment choices and ongoing evaluation of the quality of life of patients [13].

## Conclusions

According to this study, the type of clinic affects patient satisfaction. The findings do, however, also imply that much of the discontent may be reduced through improved communication and level-setting expectations. High patient satisfaction should be prioritized since it enhances clinical outcomes and treatment adherence. The focus of the physician on the patient’s quality of life, which is appreciated more in dermatology consultations, particularly for those patients with chronic skin conditions, is one aspect that impacts patient satisfaction. As a result, dermatologists should regularly assess the quality of life using easy methods such as the dermatology life quality index (DLQI). Our aim is that dermatologists should continue to offer excellent care while also making an effort to establish themselves as industry leaders in patient satisfaction. It is possible to convince insurers that our area of expertise is unique by relating their happiness with medical care to patients’ quality of life.
